# Gender bias and sex-based differences in health care efficiency in Polish regions

**DOI:** 10.1186/s12939-016-0501-y

**Published:** 2017-01-11

**Authors:** Błażej Łyszczarz

**Affiliations:** Department of Public Health, Faculty of Health Sciences, Nicolaus Copernicus University in Toruń, ul. Sandomierska 16, Bydgoszcz, 85-830 Poland

**Keywords:** Health production, Gender bias, Life expectancy, Health care efficiency, Doctor density, Panel data

## Abstract

**Background:**

Health differences between sexes are relatively well recognized, though less is known about the specificity of women's and men's health responsiveness to medical care. Applying data from Polish regions, this study identifies sex-based differences in medical care efficiency and investigates the reasons for these disparities in the gender bias context.

**Methods:**

The study estimates sex-specific health production functions for regional data from Poland (1999–2013). Using panel-data regression, male and female life expectancies at ages 0, 15, 30, 45, 60 and 65 are regressed on a set of socioeconomic factors, with the primary interest in medical care proxied by doctor density.

**Results:**

The results show that in Poland the association between life expectancy and doctor density was positive for both men and women; however, the coefficients for medical care were insignificant for those at birth and at the age of 30 for both sexes. The magnitude of health care for longevity was higher for men comparing to women at every age, though the difference between sexes was not statistically significant. The sex-based disparities in medical care efficiency were more pronounced at younger ages and they diminished with age. The inspection of data on the health system in Poland shows that male patients seemed to be in an advantageous position: the mean reimbursement per service for men was higher in most medical care areas; men reported less problems with access to health care; and their mortality trend exhibited more favorable evolution over time. Additionally, the association between other socioeconomic factors and health also differed across sexes, and several of these factors were more important for life expectancy than health care.

**Conclusion:**

Polish medical care suffers from gender bias, which possibly makes men more responsive to medical care. The disparities in the operation of medical care in Poland should be challenged to achieve more equal access to services between sexes and possibly to gain more health from the treatment of female patients.

**Electronic supplementary material:**

The online version of this article (doi:10.1186/s12939-016-0501-y) contains supplementary material, which is available to authorized users.

## Background

The last decades have witnessed remarkable progress in recognizing and understanding sex-based differences in health and medical care utilization [1–3]. A sex-based approach to medicine is important because men’s and women’s health responds differently to particular factors and identifying sex-specific health needs may be essential to increase both efficiency and equity in medical care. A notable area of interest in this field of health research is gender bias. The term 'gender bias' in medicine refers to clinically unjustified differences in diagnosis and treatment of male and female patients and most of the evidence suggests that women experience more difficulties for this reason [1, 4]. The disparities between sexes in medicine arise from many factors: differences in the way that men and women perceive and report their illness and symptoms, misjudgment of a woman's health risk, misperception of risks and benefits of particular medical procedures, unconscious prejudice and explicit discrimination of women, and cultural biases [5]. Consequently, gender bias in medicine results in sex-based inequities in access to and utilization of health services, leading to probable differences in men's and women's health responsiveness to medical care.

Although there are several aggregate-level studies concerned with estimating the association between medical care and the health of women and men, so far they have failed to arrive at unambiguous conclusions about which sex is more responsive to medical care. It is unclear whether men benefit from health care more than women do, as argued by some authors [6–9], or whether the opposite is correct [10–13]. This ambiguity of results shows that the associations involved are contextual and that the conclusions drawn from previous studies vary considerably and depend heavily upon the selection of variables and estimation techniques (Additional file 1 provides a summary of relevant studies on the topic). Moreover, very little is known about the reasons for sex differences in the role of medical care in health production. In fact, in the earlier studies, the data constraints prevented researchers from untangling complex sex-specific relationships and only allowed for speculations on potential reasons for these differences [6–14].

This study addresses the problem of differences in the responsiveness of women’s and men’s health to medical care by applying a health production framework to Polish regional data. Unlike in the previous studies [6–14], here, the analysis was not limited solely to identifying the sex-based differences, we also attempted to examine whether these disparities originated from gender bias in the health system’s capabilities to meet the patients’ needs. The differences between men and women were identified by regressing sex- and age-specific life expectancies (LEs) on doctor densities, controlling for several socioeconomic confounders. Then, we explained these differences using data on Polish medical care and showed that men's higher responsiveness to health care in Poland may result from their privileged position with respect to health system operation. Therefore, the aim of this study was to identify sex differences in medical care efficiency and to examine whether the disparities identified were attributable to gender bias in Polish health care. For this purpose, we used panel data regression for dataset of 16 Polish regions in the period 1999–2013 combined with data on sex-based disparities in various areas of health system in Poland.

## Methods

### Health production function

The determinants of health are frequently investigated within the health production framework, in which socioeconomic and environmental factors are inputs in the production of health outcomes [9, 11, 12]. The health production theory is built upon Grossmann's demand for health model wherein health-related choices are explained in terms of utility gains obtained from various health states [15]. Individuals as health producers maximize health subject to budget constraints, genetic endowment and individual responses to health behaviors. The concept of health production can be specified in terms of the following functional relationship:

*H* = *f*(*X*),

where *H* is a measure of health status and is the output of the production process, and *X* is a vector of inputs including factors, such as health care utilization, income, education, health-related lifestyle, environmental factors and initial health status. In the present study, the main focus of the analysis was on identifying the relationship between medical care and health outcomes, whereas the other determinants were confounding factors.

### Models

We used panel data regression to test the association between health determinants and sex-specific LEs. Two of the most common approaches used in estimating panel data equations are the fixed effects (FE) model and random effects (RE) model [16]. Panel diagnostic tests (F, Breusch-Pagan and Hausman tests) were used to choose between the one-way FE and RE models. For the same reasons as in the study of Canadian regions [17], the two-way FE model was not used; the inclusion of time dummy variables into the models captured much of the variance in the dependent variables and obscured the underlying causes of the trend.

The specification of the models estimated was given by the following equation:

$$ {H}_{it} = a + {\alpha}_i + \beta {M}_{it-k} + \gamma E{L}_{it-k} + {\varepsilon}_{it} $$

where *H*_*it*_ is a sex- and age-specific life expectancy (LE) in region *i* and in year *t*, *a* is a constant term, *α*_*i*_ is a fixed effect for region *i* (FE models only), *M*_*it-k*_ is an indicator of medical care resources employed in region *i* and *k* years prior to year *t*, *EL*_*it-k*_ is a vector of environmental and lifestyle factors in region *i*, lagged for *k* years and *ε*_*it*_ is an error term. *β* and *γ* are vectors of the coefficients to be estimated. All of the variables were expressed in natural logarithms, so the coefficients could be interpreted in terms of constant elasticities.

The independent variables were used in a lagged form, which allowed incorporation of the fact that the impact of factors affecting health manifests itself after some time, not instantaneously. Due to the short time span of the dataset, we used lags from one to three years. Possibly, the cumulative effect of factors affecting health manifests itself after more than three years; nonetheless, it is preferred to use short lags instead of none. The dependent variables referred to the period 2002–2013, and the explanatory variables covered the years 1999–2010 for all but one variable. Altogether, data on 16 regions and 12 years (192 observations) in each of the models was used. The completeness of the dataset was satisfactory, and only one variable contained missing data^1^.

### Variables

In our models, we used sex- and age-specific measures of health status as a dependent variable and a set of socioeconomic and environmental covariates. Additional file 2 shows the names, definitions and descriptive statistics of the variables used.

The population health status was proxied by LE, which is one of the common measures of health used in aggregate-level analyses [10, 18]. LE is the average number of years that a person at a certain age is expected to live, provided the existing age-specific mortality rates in the population persist in the future. In the present study, sex-specific LEs at ages 0, 15, 30, 45, 60 and 65 were used.

We distinguished among three categories of inputs in health production: medical care resources, environmental factors and lifestyle factors. The medical care resources were measured using doctor density. The number of doctors reflects the health care supply; an insufficient availability of physicians leads to long waiting times to receive treatment and, therefore, makes health care less effective [18]. Although doctors represent only one group of resources used in the medical sector, it is argued that doctors characterize the key input in health care production, leading diagnostic processes, deciding on treatments and typically being responsible for technological changes [19]. The physical environment was proxied by the emission of sulfur dioxide. Social and economic phenomena affecting health include a number of factors, of which the most prominent are education and income. Education was measured by the share of population with tertiary education. The measure of economic conditions that people face was per capita disposable income. Other socioeconomic factors of interest were working conditions, proxied by the percentage of workforce employed in the service sector, and housing conditions, measured with the average useable floor space of dwelling. Because of the lower data availability for the housing variable, it was the only covariate used in an unlagged form. Two lifestyle factors were included in the analysis: alcohol and tobacco consumption and physical activity. The former was proxied by per capita real expenditure on tobacco and alcohol together, whereas physical activity was represented by the number of sport clubs members^2^.

Three of the variables used—educational attainment, services employment and sport clubs members—were broken down according to sex. This approach allowed for obtaining more precise results because in all of these dimensions, the differences between men and women in Poland are noticeable (see Additional file 2 for details).

### Estimation strategy

The empirical strategy applied here was based on estimating the models with various lag lengths for covariates and choosing the most appropriate lags based on the values of Akaike information criterion (AIC), which is commonly used for this purpose [20]. Multicollinearity among the independent variables was checked for, and collinearity problems (simple correlation coefficient exceeding 0.5) were detected between income and education as well as income and tobacco and alcohol expenditure. To test whether the multicollinearity causes instability of the coefficients, alternative specifications were estimated with the troublesome variables omitted. To further examine the robustness of the results, the final specifications were compared with several models based on the other estimation method and alternative lag patterns.

### Data sources

The data used in estimating health production models was obtained from the Local Data Bank [21], which is an online regional database published by the Central Statistical Office of Poland. Due to data availability, the period of the analysis was limited to years 1999–2013; all 16 Polish regions were included with the same number of observations making the panel used balanced. The data on the sex-specific aspects of health system operation were taken from various sources and the detailed references for these sources are provided in the respective parts of the paper.

## Results

### Health status and health care in Poland

In 2013, the average LE in Poland reached 81.2 years for females and 73 years for males, considerably lower than the OECD mean values (83.1 years and 77.8 years, respectively). Poland experienced substantial health improvements in the 1990s, however, in the 2000s, the LE dynamics diminished and was lower than in most OECD countries. A distinctive characteristic of Poland is a sizeable LE difference between sexes. On average, in 2013 women in Poland had an 8.2 year higher LE than men, and the only OECD country with a wider LE sex gap was Estonia (see Additional file 3 for details). Poland spends 6.4% of its gross domestic product (GDP) on health care, placing far behind the average of 8.9% in the OECD (data for 2013). The number of medical professionals is also low compared to other countries of the region. The density of doctors was 2.2 per 1.000 population in Poland, whereas the same measure for Hungary, Estonia, Slovakia and the Czech Republic ranged from 3.2 to 3.7 (see Additional file 4 for details) [22].

### Health production function estimates

Table 1 presents the estimates of the models explaining the determinants of LEs for both sexes and each of the six ages. The models' specifications were chosen based on the values of AIC and panel diagnostic tests. Based on these criteria, the specifications using the random effects models with explanatory variables lagged for three years proved to be the best choice.

**Table 1:** Estimates of health production functions for men and women in Polish regions (1999–2013)

	Dependent variable: life expectancy at ages 0, 15, 30, 45, 60, 65											
	Females						Males					
Independent variables	F_0	F_15	F_30	F_45	F_60	F_65	M_0	M_15	M_30	M_45	M_60	M_65
Constant	3.6366^c^ (0.0719)	3.3800^c^ (0.0746)	2.9284^c^ (0.0946)	2.3200^c^ (0.1263)	1.5536^c^ (0.1918)	1.1596^c^ (0.2368)	3.1964^c^ (0.0896)	2.8291^c^ (0.1084)	2.1739^c^ (0.1398)	1.4776^c^ (0.1740)	−0.0226 (0.2616)	−0.7311^b^ (0.3104)
Doctor density	0.0034 (0.0029)	0.0065^b^ (0.0030)	0.0064^a^ (0.0039)	0.0117^b^ (0.0052)	0.0212^c^ (0.0077)	0.0249^c^ (0.0095)	0.0066 (0.0041)	0.0121^b^ (0.0049)	0.0105 (0.0064)	0.0175^b^ (0.0082)	0.0254^b^ (0.0108)	0.0345^b^ (0.0128)
Education	0.0113^c^ (0.0019)	0.0114^c^ (0.0025)	0.0151^c^ (0.0025)	0.0218^c^ (0.0034)	0.0406^c^ (0.0051)	0.0566^c^ (0.0063)	0.0071^b^ (0.0029)	0.0071^b^ (0.0035)	0.0092^b^ (0.0046)	0.0177^c^ (0.0059)	0.0351^c^ (0.0078)	0.0411^c^ (0.0092)
Income	0.0103^c^ (0.0038)	0.0134^c^ (0.0039)	0.0164^c^ (0.0050)	0.0176^c^ (0.0066)	0.0072 (0.0100)	0.0094 (0.0122)	0.0446^c^ (0.0051)	0.0531^c^ (0.0061)	0.0645^c^ (0.0080)	0.0778^c^ (0.0101)	0.0287^b^ (0.0138)	0.0227 (0.0164)
Services employment	0.0120^b^ (0.0052)	0.0129^b^ (0.0030)	0.0157^b^ (0.0070)	0.0186^b^ (0.0093)	0.0228 (0.0140)	0.0228 (0.0171)	0.0105^a^ (0.0062)	0.0121 (0.0074)	0.0196^b^ (0.0098)	0.0162 (0.0125)	0.0011 (0.0165)	0.0073 (0.0194)
Housing conditions	0.1560^c^ (0.0198)	0.1637^c^ (0.0206)	0.2038^c^ (0.0261)	0.2620^c^ (0.0348)	0.3390^c^ (0.0529)	0.3776^c^ (0.0653)	0.1881^c^ (0.0249)	0.2078^c^ (0.0302)	0.2766^c^ (0.0388)	0.3259^c^ (0.0483)	0.6222^c^ (0.1428)	0.7427^c^ (0.0869)
Pollution	−0.0013 (0.0008)	−0.0017^b^ (0.0008)	−0.0023^b^ (0.0011)	−0.0028^a^ (0.0014)	−0.0043^a^ (0.0022)	−0.0057^b^ (0.0027)	−0.0020^a^ (0.0011)	−0.0024^a^ (0.0013)	−0.0034^b^ (0.0017)	−0.0054^b^ (0.0021)	−0.0120^c^ (0.0031)	−0.0124^c^ (0.0037)
Alcohol and tobacco	−0.0081^c^ (0.0029)	−0.0086^c^ (0.0031)	−0.0106^c^ (0.0039)	−0.0136^c^ (0.0052)	−0.0165^b^ (0.0077)	−0.0248^c^ (0.0095)	−0.0141^c^ (0.0041)	−0.0137^c^ (0.0049)	−0.0133^b^ (0.0064)	−0.0191^b^ (0.0082)	−0.0198^a^ (0.0108)	−0.0184 (0.0127)
Physical activity	0.0041^c^ (0.0015)	0.0058^c^ (0.0016)	0.0070^c^ (0.0020)	0.0096^c^ (0.0027)	0.0136^c^ (0.0040)	0.0137^c^ (0.0049)	0.0005 (0.0025)	−0.0005 (0.0030)	−0.0039 (0.0053)	0.0006 (0.0050)	0.0072 (0.0066)	0.0128 (0.0078)
F-test for panels	27.16 (*p* < 0.001)	33.27 (*p* < 0.001)	32.57 (*p* < 0.001)	26.83 (*p* < 0.001)	18.25 (*p* < 0.001)	14.56 (*p* < 0.001)	69.11 (*p* < 0.001)	76.25 (*p* < 0.001)	68.45 (*p* < 0.001)	54.81 (*p* < 0.001)	33.59 (*p* < 0.001)	27.10 (*p* < 0.001)
Breusch-Pagan test	243.62 (*p* < 0.001)	259.10 (*p* < 0.001)	246.38 (*p* < 0.001)	204.23 (*p* < 0.001)	169.40 (*p* < 0.001)	154.54 (*p* < 0.001)	684.17 (*p* < 0.001)	698.70 (*p* < 0.001)	674.56(*p* < 0.001)	595.05 (*p* < 0.001)	370.52 (*p* < 0.001)	288.81 (*p* < 0.001)
Hausman test	9.95 (*p* = 0.269)	10.29 (*p =* 0.245)	10.45 (*p =* 0.235)	10.47 (*p =* 0.233)	9.89 (*p =* 0.273)	9.71 (*p =* 0.286)	2.83 (*p =* 0.945)	2.99 (*p =* 0.935)	2.90 (*p =* 0.940)	3.81 (*p =* 0.874)	10.56 (*p =* 0.228)	11.20 (*p =* 0.191)

Source: own calculations based on [21]. Notes: Each model built with 192 observations. All the variables are expressed in natural logarithms. The values in parenthesis for variables estimates are standard errors. Models are estimated using random-effects method and Nerlove's technique is used in the generalized least squares procedure. All the covariates except "Housing conditions" are lagged for three years. The variables for education, employment in services and physical activity are sex-specific. ^a^, ^b^, ^c^ - coefficients significant at the 0.1, 0.05 and 0.01 levels, respectively

**Fig. 1:**
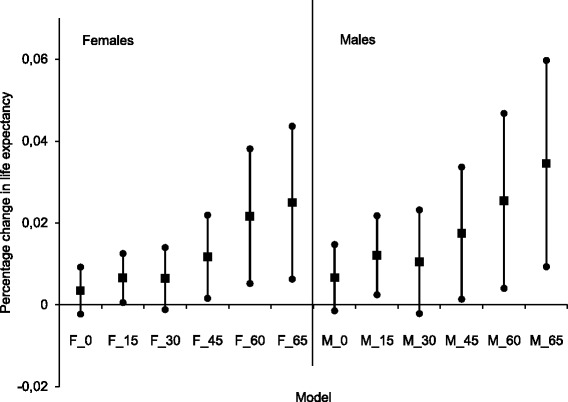
Sex- and age-specific elasticities of life expectancy with respect to health care. Source: own calculations based on [21]. Notes: Point and 95% confidence interval estimates

The results show that the association between medical care proxied by doctor availability and LE varied with sex and age, though the differences between men and women for particular ages were not significant (Fig. 1).

The association between doctor density and LE was positive in every estimated model; however, in the equations explaining the health of those at birth and at the age of 30, the coefficients were insignificant (at 0.95 confidence level) for both sexes. The magnitude of health care was higher for men at every age, with the highest differences between sexes characterizing the young. The value of the coefficient at birth was almost twice as high for males, and the respective difference between men and women was markedly smaller for the elderly. A 100% growth in doctor density was associated with an increase in LE at birth of 0.66% for men (5.6 months of LE gained) and 0.34% for women (3.2 months); the respective values for men and women at 65 years were 3.45% (6 months) and 2.49% (5.6 months). These results show that health gains from higher physician availability were modest, and other factors contributed more to increasing the longevity of the Polish population.

According to the estimated models, the age increment from 30 to 45 years was the life period when the importance of health care grew rapidly, and the values of the doctor density coefficients between these ages increased by 83% for women (from 0.0064 to 0.0117) and 67% for men (from 0.0105 to 0.0175) (Table 1). However, the growth of medical care importance with age differed between sexes. As Fig. 1 shows, the incremental contribution of doctor density to male's LE was stable from 30 years until 65 years, whereas the pattern for women was diversified. The increment for women was highest between 45 and 60 years and turned out to be relatively modest between 60 and 65 years.

In addition, other socioeconomic factors were associated with sex-specific LEs in Poland. Perhaps surprisingly, the most prominent factor that correlated with longevity was housing conditions. The elasticities for housing (0.156 for females and 0.188 for males at birth) were several times higher than those for other health determinants, including the elasticities for income (0.010 for females and 0.045 for males at birth) and education (0.011 for females and 0.007 for males at birth). Additionally, employment in services and physical activity were positively correlated with LE; however, these relationships were significant only for women. As expected, pollution and expenditure for stimulants were negatively correlated with health, particularly for men. Similarly to medical care, also for other covariates, sex and age differences in their associations with LE were detected. Men's health correlated more strongly with income, housing and pollution as well as alcohol and tobacco consumption, whereas women's longevity was more susceptible to education, working conditions and physical activity. Education became crucial for health at older ages, whereas the importance of income diminished for those in their 60s.

### Robustness of the results

To examine the robustness of the results, we tested various alternative model specifications. Table in Additional file 5 shows the values of sex-specific coefficients of doctor density for the models with fixed effects, covariates lagged for two and one year, unlagged covariates, an optimal combination of the lag length, and correlated covariates excluded one by one.

The use of the FE model instead of the base scenario (RE model) did not affect the results. Additionally, the exclusion of explanatory variables that could potentially cause multicollinearity problems did not alter the estimates. The one important difference is the fact that excluding education decreased the strength of the association between medical care and LE among females, which implies that education fostered the beneficial effects of health care among women. Interestingly, the results proved to be somewhat sensitive to changes in the lag length, and the estimates for the medical care seemed to be the most affected. Nevertheless, the conclusion that men benefited more from health care stands irrespective of the model applied. The picture is not that clear when the age dimension in the medical care elasticities is considered. Lagging the variables for two instead of three years elevated the coefficients notably for both men and women but only for those from 0 to 40 years; for the elderly population, the estimates were qualitatively unaffected. Moreover, the contemporary availability of doctors was not associated with the LE of women, whereas physicians' magnitude for men (but again only those from 0 to 40 years) was higher than with the variable lagged for three years.

Having the sex differences in association between doctor density and LE recognized, in subsequent section we focus on gender bias in medical care as a likely explanation for the disparities identified.

### Gender bias in health system operation

As our results show, men and women differ in many factors affecting health. Among others, the operation of the health system may contribute to sex variation in health and favor one gender over the other. The most appropriate way to account for these disparities would be to include variables that proxy sex-based differences in medical care performance directly into the regression models and draw conclusions based on estimates obtained. Unfortunately, consistent time-series regional data that illustrates gender bias in Polish health care is absent, and the following analysis rests on more general and fragmented statistics. In the subsections below we discuss gender bias in the following areas of medical care in Poland:

- reimbursement rates;
- access to health services;
- mortality dynamics;
- other areas of health system.

#### Differences in reimbursement rates

**Fig. 2:**
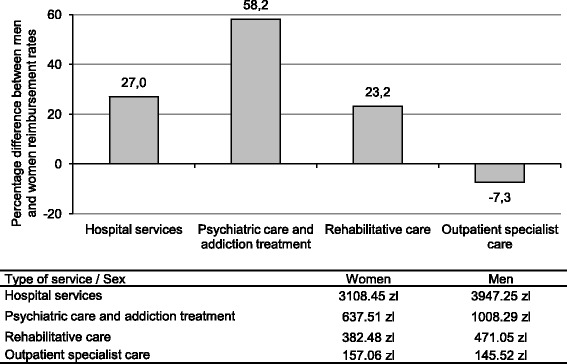
Sex differences in average provider reimbursement rates from National Health Fund in 2009. Source: [23]. Notes: The reimbursement rate for women is the reference category. A positive (negative) value of percentage difference in reimbursement rate mean that the average reimbursement for men (women) was higher. "zl" is an abbreviation for zloty – Polish currency; the average 2009 exchange rate was 3.12 zlotys per 1 US dollar. In primary care doctors are reimbursed using capitation and the amounts are not diversified by gender. National Health Fund is a state institution that finances health care benefits provided for insured population

The data show that the average reimbursement from the National Health Fund (NHF)—which is a monopolistic public third-party payer in Poland—was higher for men compared to women in hospital services, psychiatric care and rehabilitative care. The reimbursement for an average service provided to men in 2009 in these areas of health care was higher by 23.2 to 58.2%. The single sector where the reimbursement for women was higher was outpatient specialist care, with only 7.3% difference (Fig. 2). It is worth noting that the outpatient care, which was the only sector with higher average expenditure for women, was the one with the lowest rates of the four sectors. Thus, altogether the spending for men was noticeably higher.

With only aggregated data available, it is difficult to determine the reasons for men’s higher reimbursement rates. The difference may arise from males’ greater health needs, which lead to higher payments or, alternatively, the disparity is due to gender bias in the health system operation. Regarding the first explanation, it is argued that men in Poland neglect their health and do not seek medical attention until their health status worsens severely [24]. Therefore, because men use health services experiencing conditions at more developed stages and later in their life course, their treatment is more expensive. This reasoning is unlikely, however; in fact, both subjective and objective health measures from Poland show that these are women who experience more dramatic deterioration of health with aging.

**Fig. 3:**
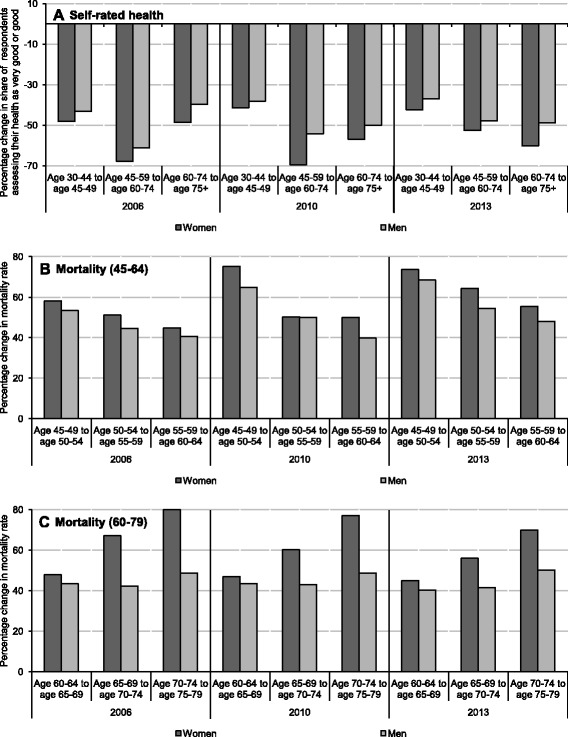
Sex differences in self-rated health status and mortality rates between populations at various age. Source: own calculations based on [25]. Notes: Panel **a** shows percentage decline in share of respondents rating their health as very good or good with each consecutive age group. Panels **b** and **c** show percentage increase in mortality rates (number of deaths per 100.000 population) with each consecutive age group

The share of men reporting very good or good health status declines with age at a lower rate than in the case of women (Fig. 3a). The proportion of respondents assessing their health positively falls by 42.3% when one compares women at age groups 30–44 and 45–59, whereas for the same age groups among men, the decline is only 37% (data for 2013). Similar differences characterize older groups (60–74 compared to 45–59), and the sex disproportion for change in the positive assessment of health is even more evident for the eldest population (75+ years compared to those at age 60–74), with a 60.3% decline for women and 48.9% decline for men. The above data refer to the year 2013, but the tendency for men’s lower decline of self-rated health was analogous in 2006 and 2010 [25]. The subjective measures are considered to be good proxies of health status but may well reflect specific male and female attitudes towards health problems. Possibly, men report better health status due to gender roles or socioeconomic circumstances faced, which may partially explain why men’s decline in self-rated health status with age is milder than for women. Nevertheless, the sex-specific mortality data also show that women's health deteriorates with age at a faster rate than men's health. The incremental changes in sex- and age-specific mortality rates for years 2006–2013 show that for each age group, the percentage increase in number of deaths compared to younger age group was higher among women than men (Fig. 3b, c). For example, the number of deaths per 100.000 females aged 60–64 in 2013 was higher by 55.5% than the rate for women aged 55–59, whereas the corresponding difference for male age groups was only 48%. Clearly, the number of deaths is higher for men at all ages, but the relative increase of mortality with age is more substantial in the case of women for all age groups from 35–39 to 75–79, implying that in Poland, females' health deteriorates with age at a faster rate than males' health [25].

The argument that higher payments for men in Poland are related to their more dynamic health deterioration at middle and old ages is also rejected by the fact that the average reimbursement rate for hospital services, rehabilitative services and psychiatric care for men is higher than for women, not only in older population. In fact, the payments for men exceed reimbursements for women even for those under 20 years, and the gap is observed at all ages until 80–85 years depending on the service type [23]. Thus, even for young men, who do not experience health deterioration due to an accumulation of adverse lifestyle choices, the reimbursement rates for care provided are still greater than for women at corresponding ages (with the exception of outpatient specialist care).

Both sex-specific dynamics of health status deterioration and differences between men and women in age distribution of payments for services suggest that there are factors other than the sex difference in the severity of illness that bias the reimbursement gap against women. If the gap in reimbursement between men and women is due to gender bias in the health system, one would attribute it to either incentives present in third party’s payment mechanisms or to the differences in treatment that males and females receive. Regarding the first explanation, the only public payer in Poland (NHF) uses different reimbursement methods for particular services. Acute care in hospitals, ambulatory specialists care and rehabilitative services are all reimbursed using a prospective per case method, psychiatric care services are paid for using a per diem framework, and primary care providers receive capitation payments [26]. None of the above schemes contains regulations that would favor either sex, and there is no reason to expect that the sex difference in reimbursement rate results from payment regulations or NHF’s incentives towards providers. For a single specific service, providers receive the same reimbursement for men and women, and these are rather differences in the way that male and female patients are treated in terms of medical care delivered that contribute to the gap in the reimbursement. The gap may come from the fact that men obtain more accurate diagnoses and more technologically advanced services that make their treatment more expensive. The data on NHF's costs of rectal cancer treatment appear to confirm this explanation; the average cost of therapy was 15% higher for men and increased by 38% for men and only 29% for women in the period 2005–2008, showing that not only the costs of treatment but also the dynamics of these costs favored men [27].

#### Disparities in access to health services

**Fig. 4:**
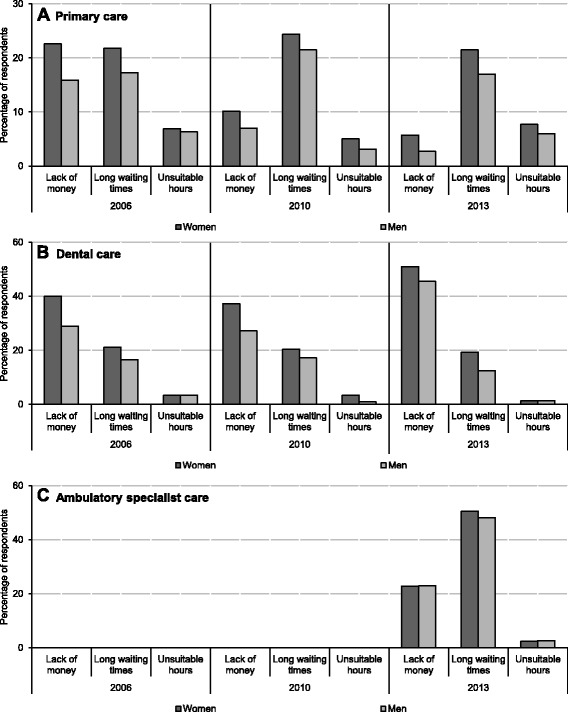
Share of respondents by reasons for not using doctors’ consultations despite being in need. Source: [28]. Notes: Data for ambulatory specialist care for 2006 and 2010 is missing. Panels **a**, **b** and **c** refer to services provided in primary care, dental care and ambulatory specialist care settings, respectively

Men's greater responsiveness to medical care can also result from their less restricted access to health services. The data from surveys over the period 2006–2013 show that more women reported difficulties in receiving care due to long waiting times, lack of money and unsuitable opening hours [28] (Fig. 4). This bias shows that the health system failed to secure equal access in the presence of economic hardships and time deficits experienced by women. Both in primary and dental care, the problems of access were more apparent among women than men throughout the period 2006–2013. Only in ambulatory specialist care were the problems of access similar for both sexes, possibly reflecting the fact that in this area of medical care, average payment was higher for women, as shown above. Also important in terms of equity, the gender bias in accessing health services was most pronounced in socially vulnerable groups—those with low income and education as well as the unemployed. However, even among those in the first income quintile, employed and well-educated, the beneficial position of men was identified [29] (see Additional file 6).

#### Differences in mortality trends

The presence of gender bias against women in the Polish health system can also be inferred from differences in mortality dynamics between men and women. The dynamics of cause-specific mortality rates between 2000 and 2013 show that the health status of men improved at a faster rate or declined at a slower rate in eight of the twelve major mortality measures, including the most common causes of death, i.e., neoplasms and diseases of circulatory system (Table 2). Various potential reasons can explain the diverging mortality trends between sexes, including the unequal treatment of men and women in the provision of health services. This inequality may be manifested in several ways. For example, the evidence on coronary heart disease suggests that men may receive better diagnoses and more effective treatment [30]. If we accept mortality rates as an indication of health care effectiveness, we observe trends supporting the hypothesis of men being in an advantageous position in cardiovascular diseases treatment in Poland. Men experienced a decline in mortality rate caused by the diseases of the circulatory system in 2000–2013 period (−1.8%), whereas the respective rate for women increased slightly (2.8%). Also mortality due to malignant neoplasms in Poland increased more among women (14.1%) than men (8.1%) in the period investigated. These sex differences in mortality trends may be an illustration of general tendencies that medicine focuses more on the conditions that strongly affect men rather than women [7]. One of the examples suggesting that men benefit more from health policy actions in Poland is difference in the dynamics of tobacco use in male and female populations. The share of daily smokers in the period 1996–2009 among men decreased from 43.8% to 30.9%, whereas the respective change for women was considerably lower—from 20.5% to 17.9% [22]. The prevalence of smoking in Poland is still higher among men; however, the sex-specific trends converge, and more rapid decline of smoking among men may result from gender bias in preventive programs that respond to men’s needs more appropriately.

**Table 2:** Cause-specific mortality rates in Poland (2000–2013)

	2000		2013		Percentage change (2000–2013)	
	Women	Men	Women	Men	Women	Men
Certain infectious and parasitic diseases (A00-B99)	0.4	0.8	0.4	0.6	0.0	−25.0
Malignant neoplasms (C00-C97)	18.5	25.9	21.1	28.0	14.1	8.1
• of stomach (C16)	1.1	2.1	0.9	1.8	−18.2	−14.3
• of trachea, bronchus and lung (C33-C34)	2.0	8.6	3.3	8.6	65.0	0.0
Diabetes mellitus (E10-E14)	1.6	1.1	2.1	1.7	31.3	54.5
Diseases of the circulatory system (I00-I99)	46.5	45.1	47.8	44.3	2.8	−1.8
• Hypertensive diseases (I10-I13)	1.4	1.0	1.2	1.0	−14.3	0.0
• Ischemic heart diseases (I20-I25)	12.5	16.7	9.3	12.0	−25.6	−28.1
• Acute myocardial infarction (I21-I22)	5.4	10.1	3.0	4.9	−44.4	−51.5
• Cerebrovascular diseases (I60-I69)	12.0	9.6	9.4	7.6	−21.7	−20.8
• Atherosclerosis (I70)	9.6	6.1	11.7	6.9	21.9	13.1
Cirrhosis of liver (K74)	0.7	1.6	0.5	0.9	−28.6	−43.8

Source: [25]. Notes: Number of deaths per 10.000 population. ICD-10 codes in parenthesis

#### Other examples of gender bias in health system

Other areas where men's situation is advantageous in the Polish health system include insurance coverage, private health financing and cultural barriers in accessing health services. The compulsory public health insurance in Poland covers a majority of the population; however, among those uninsured, women prevail. The structure of private health spending is also disadvantageous for women; voluntary private insurance coverage is biased in favor of men with their two times higher rate of insurance, whereas women tend to spend more out-of-pocket and are relatively more exposed to catastrophic health expenditures. In regard to cultural factors, due to stereotypes, female patients experience difficulties in using some procedures, including the treatment of alcohol addiction and alcohol-related diseases. Women also report discrimination and unequal treatment in using health services more frequently than men do [28, 31].

## Discussion

This study investigated the associations between doctor density and LE of men and women in Polish regions (1999–2013) controlling for several socioeconomic factors. Using panel data regression, we found that health care was more strongly associated with male's LE for all ages, although the differences between sexes were insignificant. The higher magnitude of medical care for males is in line with the results of three recent studies that used international data from OECD countries [6, 7, 9]. However, Barthold et al. [7] have confirmed the statistical significance of sex differences with respect to health care, whereas the differences reported by Asiskovitch [6], Joumard et al. [9] and herein are not that meaningful. A higher importance of health care for men has also been reported by Crémieuex et al. [8], who used regional data from Canada. On the other hand, our findings were not consistent with studies that have reported more beneficial effect of health care among women [10–13]. The ambiguity of the results reported in several studies leads to the conclusion that differences in sex-specific responses to health care are contextual and depend on the measures used as well as the time and spatial settings of the study.

We identified relatively weak associations between doctor density and LE for both men and women. Our results show that elasticity of female and male LE at birth with respect to doctor availability was 0.003 and 0.007 respectively, while most of the other studies have reported notably stronger associations; e.g. in the study using the same proxies for medical care and health status, the respective elasticies were 0.034 and 0.029 [10]. Similar differences between our estimates and others authors' results apply to models concerned with older population. These discrepancies are most likely due to he inclusion of more covariates in the models estimated here as compared to the previous studies. Controlling for more factors—e.g. housing and physical activity here—typically results in lower coefficient values for all the predictors, as shown by the example of doctor density.

Our results exhibit sex differences also in the age dimension of health production. In both men and women the age increment from 30 to 45 years was the period when the importance of health care grew rapidly; however, in the case of men the importance of doctor availability increased stably with age starting from 45 years, while for women we observed a more variable pattern. The increment for women was highest between 45 and 60 years and turned out to be relatively modest between 60 and 65 years. These results together suggest that in the male’s life-course, the period in which medical care becomes increasingly important for health comes earlier and that the growth of importance in this factor continues to be stable. On the other hand, females' health becomes more reliant on health services later in life, but as it does, the magnitude of health care increases substantially. This gender difference can possibly be explained by the fact that women tend to experience the same life-threatening diseases somewhat later in life than men do [32] and by the fact that men do not experience such crucial changes in their physiological functioning as women do in the period of their menopause.

In general, the results were robust to changes in model specification, estimation method and dynamic structure of the model. The estimates for doctor density proved to be most affected in robustness analysis; particularly the lag length affected the values of coefficients. This sensitivity of estimates calls for caution in interpreting the results but may exhibit the actual complexity of the health production dynamics. That time dimension matters for medical care-health association has been claimed by Thornton, who has shown that the impact of medical care on mortality in the US was negative with 1-year and 4-years lags used, whereas the respective effect after two and three years was positive [33].

The data on gender bias in Polish health care reported above allow for concluding that sex disparities in the efficiency of medical care may be due to unjustified disparities in the treatment of men and women. We have shown that services provided to males are reimbursed at higher rates than those delivered to females and this difference cannot be explained by men's higher health needs with aging. In fact, women's health deteriorates more rapidly in Poland as the data on self-rated health status and age-specific mortality show. Furthermore, the average payment for men is higher at virtually every age. There is also some evidence that the dynamics of sex-specific costs favored men as it is exemplified by payments for rectal cancer treatment. For comparison purposes, it should be mentioned that the sex gap in payment rates towards men is not a universal phenomenon that also characterizes other countries. In the United States, an opposite trend was observed, and the average health spending in 2004 was 32% higher for women [34], whereas recent data from three OECD countries show that 56% of health budgets was consumed by female patients [35]. Additionally, in Canada, the mean public health expenditure for females in 2012 (4.181 dollars) exceeded the average spending per male (3.563 dollars) [36]. Men's advantageous position in Polish health care is also apparent from the data on disparities in access to health services. Women experience more difficulties in using medical care due to economic and time constraints and these differences persist over time (2006–2013). Even in the better-off social groups women still report more problems in accessing care. The recent report on gender equality in the European Union (EU) seems to confirm these sex-based disparities in access to health care; Poland is the third worst performer among 28 EU member states in terms of securing equal access to services for men and women [37]. The dynamics of cause-specific mortality throughout recent years in Poland also suggests that men benefited more from improved health system operation; the sex disparity was noticeable in cardiovascular diseases and cancer mortality, among others. This discrepancy may result from better diagnosis and more effective treatment of men; however, this hypothesis is not supported by any consistent data from Poland and should be verified by additional research. Also data on public and private insurance coverage and cultural difficulties suggest that women in Poland are in a disadvantageous position in health system.

To sum up, persistent gender bias exists in the Polish health system and may play a role in differentiating women’s and men’s responsiveness to medical care. Although the figures discussed do not provide explicit evidence on causality between gender bias and sex-based differences in efficiency of medical care, we argue that the bias identified probably explains why men benefited more than women in terms of LE improvement. That health care in Poland seems to be more beneficial for men is a conclusion that should be acknowledged in designing and implementing health-enhancing actions. Nevertheless, the conclusion regarding gender bias in the health system operation should also be considered as an indication of disparities that ought to be challenged to achieve a more equal access to services between genders and possibly gain more health from the treatment of women.

### Limitations of the study

Several caveats apply to our analysis. First, the choice of the proxy for health status is problematic because LE only reflects health problems that result in death, and the measure does not give consideration to non-fatal diseases. Despite this drawback, macro-level comparisons of population health often rely on this measure because it is highly available over time and in various groups. Second, the reliability of the results is subject to the quality of the dataset used. With 192 cases, the number of observations is not very high; the 3-year lags are probably not long enough to exhibit the long-term health effects of some factors affecting health; more of the covariates could preferably be broken down by gender. The above data constraints have to be kept in mind when interpreting the results; still, the quality of the dataset used is in line with the standards of contemporary health production studies.

Third, the study is subject to limitations of aggregated analyses, including ecological fallacy. The main problem of ecological inference is reduced information due to aggregating data which may prevent from identifying parameters of interest in the original individual-level model [38]. Aggregated studies are also more often subject to confounding bias and cross-level bias. However, with the lack of appropriate disaggregated data, the approach used here seems to be the only available option for investigating the associations between health care efficiency and gender bias. Additionally, our findings are complex and exhibit some sensitivity to model specification. Although the models were robust to the choice of the estimation method and the exclusion of correlated covariates, the changes in the length of the lags for independent variables somehow affected the results. This effect, however, does not necessarily imply that the results are misleading and may reflect the real complexity of the relationships involved. It should also be remembered that the findings of this research are contextual and their generalization in other settings could be risky, especially when applied to countries that notably differ from Poland in terms of health status and socioeconomic conditions. Last, the data on gender disparities in health system operation is fragmented and does not allow to include any consistent variable into regression models. For this reason, making causal inferences on the association between gender bias and sex-specific efficiency of medical care is practically unfeasible. Still, the approach used here is a step forward; previous studies only speculated on the reasons for sex differences identified without referring to specific data on gender bias.

## Conclusion

In conclusion, medical care in Poland is more strongly associated with male's longevity at every age investigated, although the difference between sexes is not significant. Obviously, the models estimated here are too broad to precisely recognize the reasons for gender difference in responsiveness to medical care. However, the inspection of data on the health system in Poland suggests that this difference possibly arises from gender bias in the health system, namely, men's advantageous position when utilizing health services.

Bearing in mind that the results of the studies concerned with gender differences in the association of medical care and population health are puzzling, there seems to be a need for subsequent research attempting to investigate the complexity of the process. One of the promising ways to improve the knowledge on the topic is to pursue the meso-level analyses that use single-country regional data. Because the results of the research using international data are inconsistent, perhaps we can advance the understanding of the topic with single-country data. There is also potential in combining time-series cross-section data used routinely in health production estimates with detailed information on socioeconomic disparities present in health systems operation.

## Additional files

Additional file 1: Summary of results from previous studies concerned with gender-specific health production. (PDF 213 kb)
Additional file 2: Variables definitions and descriptive statistics. (PDF 135 kb)
Additional file 3: Female and male life expectancy at birth in Poland and OECD countries. (PDF 92 kb)
Additional file 4: Health expenditure and doctor availability in Poland and OECD countries. (PDF 92 kb)
Additional file 5: Robustness analysis: Estimates of coefficients for doctor density for alternative model specifications. (PDF 266 kb)
Additional file 6: Share of respondents with unmet needs due to reasons: too expensive, too far, waiting list. (PDF 12 kb)

## Abbreviations

AIC: Akaike information criterion
EU: European Union
FE: Fixed effects
LE: Life expectancy
LEs: Life expectancies
NHF: National Health Fund
OECD: Organization for Economic Co-operation and Development
RE: Random effects.

## Footnotes

1 The data on physical activity is collected biannually, and all of the missing values were imputed using linear interpolation.

2 The number of those who train in sport clubs is not a perfect measure to control for physical activity because this number does not account for informal ways of exercising; however, it is reasonable to use this proxy instead of using no information on physical activity.
