# Supplementary material for: Gender bias and sex-based differences in health care efficiency in Polish regions
Source: Int J Equity Health. 2017 Jan 11;16:8. doi: 10.1186/s12939-016-0501-y (PMC5225635; doi:10.1186/s12939-016-0501-y)
Supplement: Additional file 1: — Summary of results from previous studies concerned with gender-specific health production. (PDF 213 kb) [file 12939_2016_501_MOESM1_ESM.pdf]

Table A1. Summary of results from previous studies concerned with gender-specific health production

	Variables	Methods	Principal findings
Asiskovitch (2010) [6]	Health status: (1) LE at birth and (2) LE at age 65 years. Medical care: national health expenditure as % of GDP - (1) total; (2) public and private separately. Control variables: (1) social expenditure; (2) GDP per capita; (3) gender-specific prevalence of smoking; (4) gender-specific prevalence of overweight or obesity; (5) nitrogen oxide emission.	Hierarchical panel-data regression for 19 OECD countries (1990-2005).	Health expenditure has no statistically significant effect on either female or male LE at birth. However, strong impact of health care is identified in the case of those at age 65 for both genders. The results suggest that men's LE is more responsive to total health expenditure than women's LE (elasticities of total health expenditure: LE_F_0 = 0.004; LE_M_0 = –0.019; LE_F_65 = 0.083[a]; LE_M_65 = 0.101[a]).
Barthold et al. (2014) [7]	Health status: (1) LE at birth, (2) LE at age 40 years and (3) LE at 65 years. Medical care: cross-product between country dummy variables and national health expenditure per capita. Control variables: (1) social expenditure; (2) GDP per capita.	Ordinary least squares regressions with heteroscedasticity-consistent (White) standard errors for 27 OECD countries (1991-2007).	Health spending increases were associated with higher LE improvements for men than for women in nearly every OECD country. Elasticities of health expenditure for most efficient country (Germany): LE_F_0 = 0.090[a]; LE_M_0 = 0.155[a]; LE_F_40 = 0.161[a]; LE_M_40= 0.272[a]; LE_F_65 = 0.279[a]; LE_M_65 = 0.490[a].
Bayati et al. (2013) [38]	Health status: LE at birth. Medical care: (1) health expenditure as % of GDP; (2) one-year-olds immunized with measles-containing vaccine. Control variables: (1) GDP per capita; (2) food availability; (3) share of population employed; (4) human development index; (5) share of urban population; (6) carbon dioxide emission.	Data for the Eastern Mediterranean Region (21 countries, 1995-2007). Details on estimation method used not provided.	LE of males is affected by health expenditure, however, the impact of spending for their longevity is negative (elasticity: –0.01[b]). On the other hand, the respective elasticity for female LE is positive but insignificant and its value is very low (0.001). The association between immunization rate and LE is insignificant and very low for both genders.
Crémieux et al. (2005) [8]	Health status: (1) LE at birth; (2) LE at age 65 years; (3) gender-specific infant mortality. Medical care: (1) public and (2) private drug spending per capita; (3) non-drug health care spending. Control variables: (1) GDP per capita; (2) population	Regression models using feasible generalized least squares for panel data with correction for	Both public and private drug expenditure in Canada affect health measures positively and elasticities for men are generally higher as compared to those for women. Additionally, total non-drug health care spending per capita is statistically significant in the models concerned

	density; (3) poverty rate; (4) alcohol spending per capita; (5) gender-specific tobacco spending per capita; (6) food and non-alcoholic beverages spending.	autocorrelation and heteroscedasticity. Regional data from Canada (1981-1998).	with male health and is not significant in any of the models explaining female health. Elasticities for public drug spending: LE_F_0 = 0.009[a]; LE_M_0 = 0.011[a]; LE_F_65 = 0.012[a]; LE_M_65 = 0.029[a].
Joumard et al. (2008) [9]	Health status: (1) LE at birth; (2) LE at age 65 years; (3) PYLL.<br>Medical care: (1) total health spending per capita; (2) health practitioners.<br>Control variables: (1) tobacco, (2) alcohol and (3) fruit and vegetables consumption per capita; (4) emission of nitrogen oxide per capita; (5) share of population with at least upper secondary education; (6) GDP per capita.	Generalised least squares with correction for heteroscedasticity and first order autocorrelation with country fixed-effects using OECD dataset (23 countries, 1981-2003).	Of the several models estimated, vast majority report higher values of health care coefficient for males as compared to females, regardless of the health care resources measure used. Elasticities for health practitioners: LE_F_0 = 0.013[a]; LE_M_0 = 0.017[a]; LE_F_65 = 0.032[b]; LE_M_65 = 0.043[b]; PYLL_F = –0.089[b]; PYLL_M = –0.062. Elasticities for health spending: LE_F_0 = 0.035[a]; LE_M_0 = 0.045[a]; LE_F_65 = 0.051[a]; LE_M_65 = 0.061[a]; PYLL_F = –0.272[a]; PYLL_M = –0.300[a].
Liu et al. (2008) [17]	Health status: (1) LE at age 65 years; (2) LE at age 80 years; (3) PYLL.<br>Medical care: (1) expenditure on pharmaceuticals per capita; (2) non-pharmaceuticals health expenditure.<br>Control variables: (1) GDP per capita; (2) emission of sulphur oxide per capita; (3) gender-specific share of population who are daily smokers; (4) alcohol consumption per capita; (5) fat, (6) sugar and (7) fruit and vegetable consumption per capita.	Fixed-effects linear models with first order autoregressive disturbance with covariates lagged for five years. 14 OECD countries (1980-2001).	The effect of non-pharmaceutical spending on LE and PYLL is low and insignificant for both genders and regardless of age. The drug-specific expenditure elasticities for LE are slightly higher in the case of males while the respective PYLL coefficient is higher for females. Elasticities for drug spending: LE_F_65 = 0.033[b]; LE_M_65 = 0.037[b]; LE_F_80 = 0.029; LE_M_80 = 0.050[b]; PYLL_F = –0.063; PYLL_M = –0.042.
Nixon and Ulmann (2006) [10]	Health status: LE at birth.<br>Medical care: (1) total health expenditure per capita; (2) number of physicians per 10.000 population.<br>Control variables: (1) protein intake per capita; (2) emission of sulphur oxide per capita.	The generalised least squares method is used for fixed-effects models (15 OECD countries, 1980-1995).	The elasticities of total health expenditure are the same for men and women (0.022[a]), however, the association between physicians' density and LE is stronger for women (0.034[a]) as compared to men (0.029[a]).
Or (2000) [11]	Health status: PYLL.<br>Medical care: (1) total health expenditure per capita; (2)	The ordinary least squares method is used	The level of health expenditure affects premature mortality of women more than in the case of men.

	share of public health expenditure.<br>Control variables: (1) GDP per capita; (2) share of white-collar workers in total work force; (3) emission of nitrogen oxide per capita; (4) butter, (5) sugar and (6) alcohol consumption per capita; (7) expenditure on tobacco per capita.	to estimate models using OECD data (21 countries, 1970-1992).	According to the estimates the respective elasticity is almost five times higher for females as compared to males (PYLL_F = –0.177[a]; PYLL_M = –0.038) and the coefficient is significant only for women. The higher share of public health expenditure is beneficial for both genders with similar magnitude.
Or (2001) [12]	Health status: (1) PYLL; (2) LE at age 65 years; (3) PYLL – heart diseases; (4) PYLL – cancer.<br>Medical care: (1) number of active physicians per 1000 population; (2) share of public expenditure in total health expenditure; (3-8) set of dummy variables representing institutional characteristics of health care systems.<br>Control variables: (1) GDP per capita; (2) share of white-collar workers in total work force; (3) tobacco and (4) alcohol consumption per capita; (5) emission of nitrogen oxide per capita.	The feasible generalised least square method is used to estimate models in order to correct for cross-section heteroscedasticity and for autocorrelation specific to each unit. The data refer to 21 OECD countries (1970-1995).	Premature mortality in general as well as caused by heart diseases and cancer is affected by doctors availability stronger in the case of women than men (elasticities: PYLL_F = –0.376[a]; PYLL_M = –0.275[a]; PYLL_F_Heart = –0.658[a]; PYLL_M_Heart = –0.573[a]; PYLL_F_Cancer = –0.185[a]; PYLL_M_Cancer = –0.018). On the other hand, the elasticities for LE at the age of 65 years are the same for both genders (0.100[a]).
Poças and Soukiazis (2010) [13]	Health status: (1) LE at birth; (2) LE at the age of 65 years.<br>Medical care: doctors' (ambulatory) consultations per capita.<br>Control variables: (1) income per capita; (2) average years of education in population aged 25-64; (3) tobacco and (4) alcohol consumption.	The instrumental variables approach (two step generalized method of moments) is used for data from 17 OECD countries (1980-2004).	Arguing that health care inputs should rather be proxied by doctors' consultations instead of doctors' density or health expenditure, the study finds that health care contributes to LE at birth and at the age of 65 only in the case of women, while coefficients of men's LEs are highly insignificant. The elasticities for doctors' consultations: LE_F_0 = 0.028[a]; LE_M_0 = 0.007; LE_F_65 = 0.064[a]; LE_M_65 = 0.021.

Notes: LE – life expectancy; PYLL – potential years of life lost; LE_F_x – female life expectancy at the age of x years; LE_M_x – male life expectancy at the age of x years; PYLL_F – female potential years of life lost; PYLL_M – male potential years of life lost; GDP – gross domestic product; a, b, c - coefficients significant at the 0.01, 0.05 and 0.1 levels, respectively.
