# Supplementary material for: Gender bias and sex-based differences in health care efficiency in Polish regions
Source: Int J Equity Health. 2017 Jan 11;16:8. doi: 10.1186/s12939-016-0501-y (PMC5225635; doi:10.1186/s12939-016-0501-y)
Supplement: Additional file 2: — Variables definitions and descriptive statistics. (PDF 135 kb) [file 12939_2016_501_MOESM2_ESM.pdf]

Table A2. Variables definitions and descriptive statistics

Variable	Definition [measurement unit]	Mean		Stand. deviation		Minimum		Maximum	
Years		2002	2013	2002	2013	2002	2013	2002	2013
LE_F_0	Female life expectancy at birth [years]	78.9	81.1	0.7	1.0	77.9	80.1	80.4	82.4
LE_F_15	Female life expectancy at 15 [years]	64.6	66.6	0.6	1.0	63.6	65.5	65.9	67.8
LE_F_30	Female life expectancy at 30 [years]	49.8	51.8	0.6	0.9	48.9	50.8	51.2	52.9
LE_F_45	Female life expectancy at 45 [years]	35.4	37.2	0.6	0.8	34.6	36.3	36.7	38.3
LE_F_60	Female life expectancy at 60 [years]	22.2	23.8	0.5	0.6	21.6	23.1	23.3	24.8
LE_F_65	Female life expectancy at 65 [years]	18.1	19.7	0.4	0.5	17.6	19.2	19.1	20.6
LE_M_0	Male life expectancy at birth [years]	70.4	73.0	0.9	0.8	68.5	70.7	72.1	74.8
LE_M_15	Male life expectancy at 15 [years]	56.2	58.5	0.9	0.7	54.3	56.2	57.8	60.3
LE_M_30	Male life expectancy at 30 [years]	42.0	44.2	0.8	0.7	40.2	42.0	43.5	45.9
LE_M_45	Male life expectancy at 45 [years]	28.5	30.4	0.6	0.7	27.1	28.8	29.6	31.9
LE_M_60	Male life expectancy at 60 [years]	17.1	18.6	0.5	0.5	16.5	17.8	17.9	19.7
LE_M_65	Male life expectancy at 65 [years]	14.0	15.4	0.4	0.4	13.3	14.7	14.7	16.2
Years		1999	2010	1999	2010	1999	2010	1999	2010
Doctors' density	Number of doctors employed in health care per 1,000 population [employed persons]	2.18	2.02	0.41	0.28	1.52	1.45	2.77	2.41
Education_F	Share of female population aged 15-64 with tertiary education level [percentage]	9.3	21.8	1.6	3.1	6.7	16.8	12.2	31.3
Education_M	Share of male population aged 15-64 with tertiary education level [percentage]	7.9	15.1	1.5	2.5	5.9	12.5	11.0	23.3
Income	Average monthly real disposable income [zlotys[a] 2010]	818	1150	89	149	704	907	1027	1602
Services employment_F	Share of total female employment working in services sector [percentage]	62.8	70.2	7.7	5.6	50.8	61.8	73.8	78.9
Services employment_M	Share of total male employment working in services sector [percentage]	40.8	42.7	5.2	5.1	31.9	36.3	49.2	55.6
Housing conditions[b]	Average useable floor space of dwelling [square meters]	68.8	73.3	4.4	4.3	63.9	67.5	76.0	80.2
Pollution	Emission of sulphur dioxide (SO_2) per square kilometre [tones/year]	4.06	1.78	5.01	1.95	0.33	0.16	16.5	7.50
Alcohol and tobacco	Average monthly household real[c] expenditure on alcohol and tobacco [zlotys[a] 2010]	27.4	26.6	4.0	5.6	19.4	18.1	32.0	36.2
Physical activity_F	Female members of sport clubs per 1,000 population	7.7	11.1	2.1	2.4	4.3	8.1	10.9	15.8
Physical activity_M	Male members of sport clubs per 1,000 population	23.9	35.0	6.1	7.2	13.9	26.9	34.5	54.8

Source: own calculations based on [20]. Notes: a – the zloty is the Polish monetary unit; the average 1999 exchange rate was 3.97 zlotys per 1 US dollar; the average 2010 exchange rate was 3.02 zlotys per 1 US dollar; b - due to data limitations the variable "Housing" is not used in a lagged form, thus, the data provided refers to years 2002 and 2013 not to years 1999 and 2010 as in the case of other covariates; c - to incorporate the fact that the prices of tobacco were

rising more rapidly than the prices of other goods and services the specific consumer price index (CPI) for alcohol and tobacco is used to deflate the expenditures in this category instead of general CPI index.
