# Supplementary material for: Gender bias and sex-based differences in health care efficiency in Polish regions
Source: Int J Equity Health. 2017 Jan 11;16:8. doi: 10.1186/s12939-016-0501-y (PMC5225635; doi:10.1186/s12939-016-0501-y)

Figure A1. Female and male life expectancy (LE) at birth in Poland and OECD countries

Panels A and B show dynamics of female and male life expectancy in Poland, four other countries of the region and OECD average. Panel C shows gender gap in life expectancy in OECD countries. Source: [21].

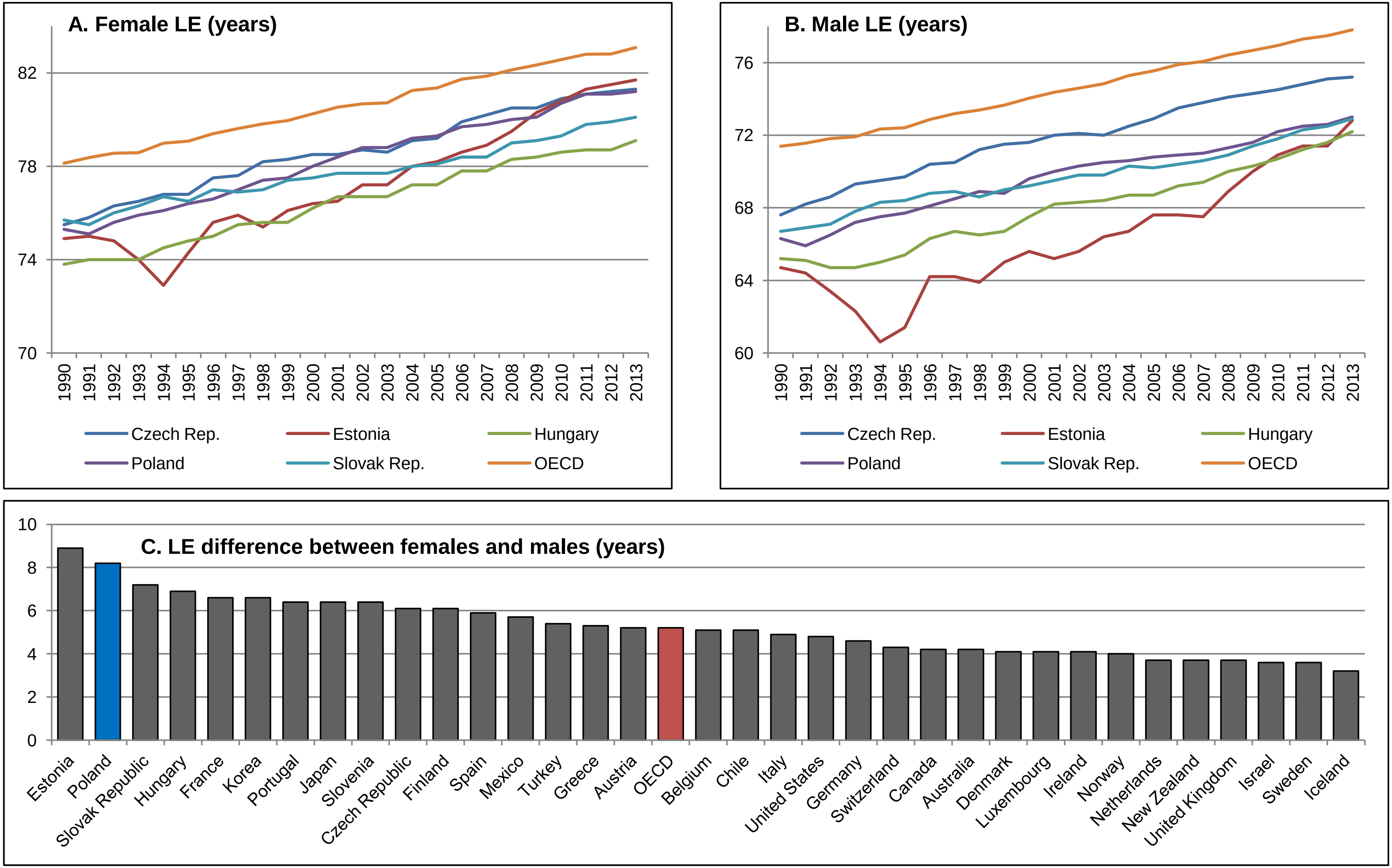

Supplement: Additional file 3: — Female and male life expectancy at birth in Poland and OECD countries. (PDF 92 kb) [file 12939_2016_501_MOESM3_ESM.pdf]
