# Supplementary material for: Gender bias and sex-based differences in health care efficiency in Polish regions
Source: Int J Equity Health. 2017 Jan 11;16:8. doi: 10.1186/s12939-016-0501-y (PMC5225635; doi:10.1186/s12939-016-0501-y)

Figure A2. Health expenditure and doctor availability in Poland and OECD countries

Panel A shows dynamics of current health expenditure in Poland, four other countries of the region and OECD average. Panel B shows dynamics of doctor density in the same countries. Panel C shows annual growth rate of health expenditure in OECD countries. Source: [21].

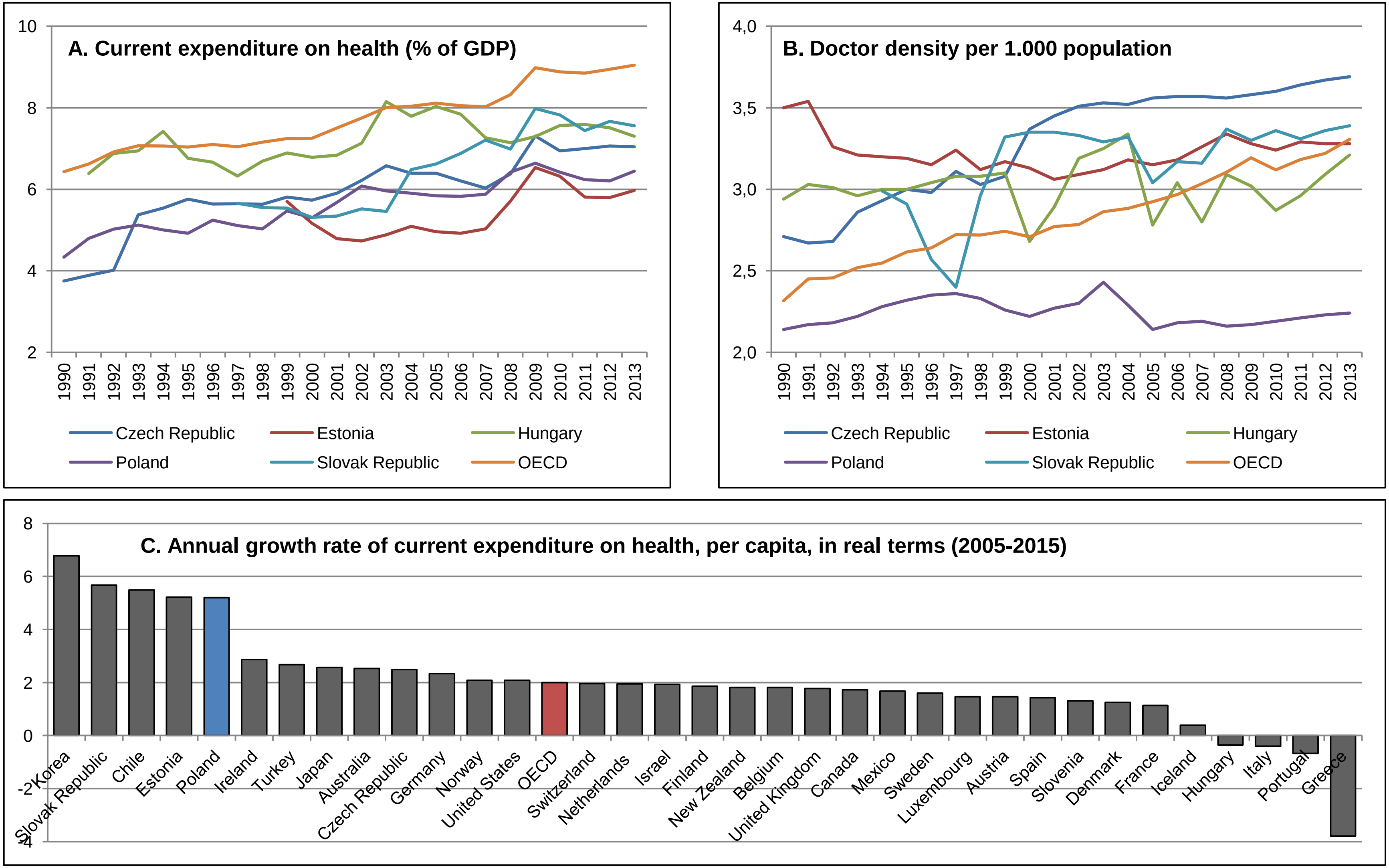

Supplement: Additional file 4: — Health expenditure and doctor availability in Poland and OECD countries. (PDF 92 kb) [file 12939_2016_501_MOESM4_ESM.pdf]
