# Supplementary material for: Gender bias and sex-based differences in health care efficiency in Polish regions
Source: Int J Equity Health. 2017 Jan 11;16:8. doi: 10.1186/s12939-016-0501-y (PMC5225635; doi:10.1186/s12939-016-0501-y)
Supplement: Additional file 5: — Robustness analysis: Estimates of coefficients for doctor density for alternative model specifications. (PDF 266 kb) [file 12939_2016_501_MOESM5_ESM.pdf]

Table A3. Robustness analysis: Estimates of coefficients for doctor density for alternative model specifications

	Dependant variable: life expectancy at ages 0, 15, 30, 45, 60, 65											
	Females						Males					
	F_0	F_15	F_30	F_45	F_60	F_65	M_0	M_15	M_30	M_45	M_60	M_65
Base model	0.0034	0.0065**	0.0064*	0.0117**	0.0212***	0.0249***	0.0066	0.0121**	0.0105	0.0175**	0.0254**	0.0345**
	(0.0029)	(0.0030)	(0.0039)	(0.0052)	(0.0077)	(0.0095)	(0.0041)	(0.0049)	(0.0064)	(0.0082)	(0.0108)	(0.0128)
One-way fixed effects model	0.0033	0.0063	0.0061	0.0115*	0.0216**	0.0260**	0.0073	0.0129*	0.0113	0.0179	0.0251*	0.0346**
	(0.0033)	(0.0038)	(0.0045)	(0.0063)	(0.0083)	(0.0101)	(0.0059)	(0.0068)	(0.0096)	(0.0126)	(0.0149)	(0.0147)
Model with covariates lagged for two years	0.0055**	0.0083***	0.0107***	0.0135***	0.0206***	0.0236***	0.0152***	0.0201***	0.0214***	0.0316***	0.0250**	0.0258**
	(0.0027)	(0.0028)	(0.0035)	(0.0047)	(0.0073)	(0.0088)	(0.0038)	(0.0046)	(0.0060)	(0.0077)	(0.0102)	(0.0119)
Model with covariates lagged for one year	0.0020	0.0047*	0.0066**	0.0059	0.0068	0.0056	0.0192***	0.0254***	0.0302***	0.0406***	0.0242**	0.0231**
	(0.0025)	(0.0026)	(0.0033)	(0.0044)	(0.0067)	(0.0083)	(0.0039)	(0.0049)	(0.0063)	(0.0079)	(0.0095)	(0.0111)
Model with unlagged covariates	–0.0012	0.0026	0.0039	0.0027	0.0009	–0.0004	0.0166***	0.0246***	0.0295***	0.0388***	0.0085	0.0082
	(0.0027)	(0.0028)	(0.0036)	(0.0047)	(0.0071)	(0.0089)	(0.0041)	(0.0050)	(0.0064)	(0.0083)	(0.0105)	(0.0124)
Model with optimized lag structure	‡0.0039	†0.0050*	†0.0064*	‡0.0167***	‡0.0227***	‡0.0236***	§0.0066	§0.0121**	§0.0105	§0.0175**	†0.0221**	†0.0191*
	(0.0025)	(0.0026)	(0.0033)	(0.0048)	(0.0071)	(0.0084)	(0.0041)	(0.0049)	(0.0064)	(0.0082)	(0.0091)	(0.0108)
Model without education variable	0.0020	0.0051	0.0045	0.0090	0.0165*	0.0186*	0.0066	0.0121**	0.0105	0.0176**	0.0246**	0.0336**
	(0.0031)	(0.0033)	(0.0041)	(0.0056)	(0.0088)	(0.0111)	(0.0042)	(0.0049)	(0.0065)	(0.0084)	(0.0113)	(0.0134)
Model without income variable	0.0031	0.0061*	0.0058	0.0112**	0.0210***	0.0247***	0.0056	0.0107*	0.0087	0.0152	0.0245**	0.0339***
	(0.0030)	(0.0031)	(0.0039)	(0.0052)	(0.0077)	(0.0095)	(0.0048)	(0.0058)	(0.0074)	(0.0093)	(0.0109)	(0.0128)
Model without alcohol and tobacco variable	0.0025	0.0055*	0.0051	0.0101*	0.0193**	0.0220***	0.0048	0.0103**	0.0088	0.0151*	0.0227**	0.0320**
	(0.0029)	(0.0031)	(0.0039)	(0.0052)	(0.0078)	(0.0095)	(0.0042)	(0.0049)	(0.0064)	(0.0082)	(0.0108)	(0.0127)

Notes: Each model built with 192 observations. All the variables are expressed in natural logarithms. The values in parenthesis for variables estimates are standard errors. Optimal lag structure is based on Akaike Information Criteria values. Nerlove's technique is used in the generalised least squares procedure for the RE models. Heteroscedasticity and autocorrelation consistent standard errors are used in the FE model. *, **, *** - coefficients significant at the 0.1, 0.05 and 0.01 levels, respectively; †, ‡, § - one, two and three years lags, respectively.
