# Supplementary material for: Gender bias and sex-based differences in health care efficiency in Polish regions
Source: Int J Equity Health. 2017 Jan 11;16:8. doi: 10.1186/s12939-016-0501-y (PMC5225635; doi:10.1186/s12939-016-0501-y)

Figure A3. Share of respondents with unmet needs due to reasons: too expensive, too far, waiting list. Source: [28]

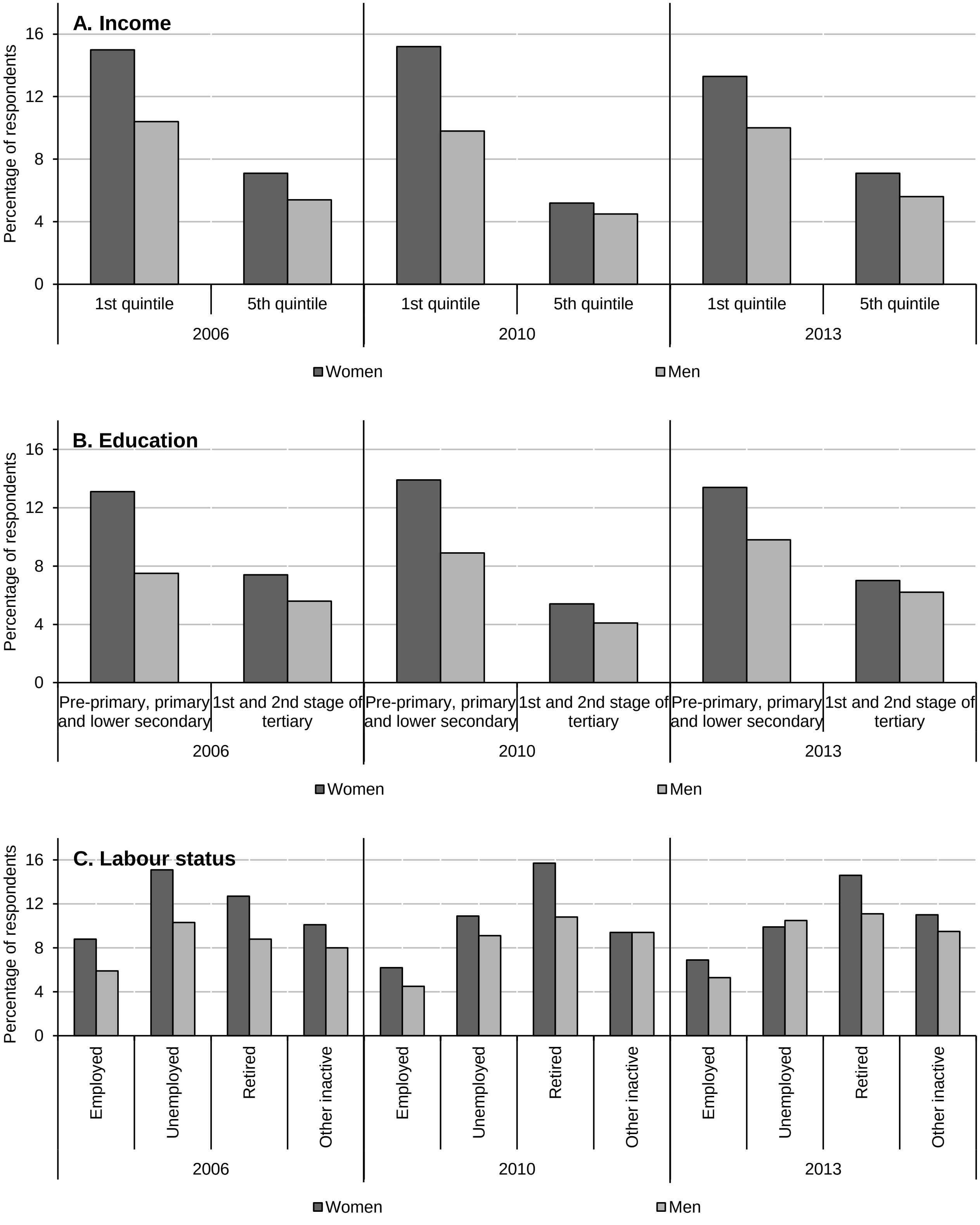

Supplement: Additional file 6: — Share of respondents with unmet needs due to reasons: too expensive, too far, waiting list. (PDF 12 kb) [file 12939_2016_501_MOESM6_ESM.pdf]
